# Appetite Loss and Anorexia of Aging in Clinical Care: An ICFSR Task Force Report

**DOI:** 10.14283/jfa.2022.14

**Published:** 2022-02-22

**Authors:** Philipe de Souto Barreto, M. Cesari, J. E. Morley, S. Roberts, F. Landi, T. Cederholm, Y. Rolland, B. Vellas, R. Fielding

**Affiliations:** 1 Gérontopôle de Toulouse, Institut du Vieillissement, Centre Hospitalier-Universitaire de Toulouse, 37 Allées Jules Guesde, 31000 Toulouse, France; 2 CERPOP, Inserm 1295, Université de Toulouse, UPS, Toulouse, France; 3 IRCCS Istituti Clinici Scientifici Maugeri, University of Milan, Milano, Italy; 4 Division of Geriatric St Louis University Medical School, St Louis, USA; 5 Energy Metabolism Laboratory, Jean Mayer USDA Human Nutrition Research Center on Aging, Boston, MA USA; 6 Fondazione Policlinico Universitario “Agostino Gemelli” IRCCS, Rome, Italy; 7 Uppsala University, Department of Public Health and Caring Sciences, Uppsala, Sweden; 8 Nutrition, Exercise Physiology, and Sarcopenia Laboratory, Jean Mayer USDA, Human Nutrition Research Center on Aging at Tufts University, Boston, MA USA

**Keywords:** Anorexia, nutritional status, older adults, frailty, intrinsic capacity

## Abstract

Appetite loss/anorexia of aging is a highly prevalent and burdensome geriatric syndrome that strongly impairs the quality of life of older adults. Loss of appetite is associated with several clinical conditions, including comorbidities and other geriatric syndromes, such as frailty. Despite its importance, appetite loss has been under-evaluated and, consequently, under-diagnosed and under-treated in routine clinical care. The International Conference on Frailty and Sarcopenia Research (ICFSR) Task Force met virtually on September 27th 2021 to debate issues related to appetite loss/anorexia of aging. In particular, topics related to the implementation and management of appetite loss in at-risk older adult populations, energy balance during aging, and the design of future clinical trials on this topic were discussed. Future actions in this field should focus on the systematic assessment of appetite in the care pathway of older people, such as the Integrated Care for Older People (ICOPE) program recommended by the World Health Organization. Moreover, clinical care should move from the assessment to the treatment of appetite loss/anorexia. Researchers continue to pursue their efforts to find out effective pharmacologic and non-pharmacologic interventions with a favorable risk/benefit ratio.

## Introduction

**A**ppetite is thought to be a major determinant of nutritional status during aging. Its loss, ie, anorexia of aging, is a common clinical condition in older adulthood (1–6). Indeed, the prevalence of appetite loss is high, occurring in approximately 15%-30% of community-dwelling older people and even higher in clinical settings such as hospitals and nursing homes (7, 8). Loss of appetite is associated with a myriad of clinical conditions during aging, including comorbidities and major geriatric syndromes, such as depression, apathy, sarcopenia, malnutrition and cachexia, and frailty (8–13). Despite the importance of appetite loss for the care of older adults, the assessment and management of this condition remain suboptimal in clinical settings, being characterized by its under-diagnosis, poor consideration of its burdening effects, and consequent under-treatment. This is partly explained by an ageist approach to the anorexia of aging, since this condition has often been erroneously assumed to be a normal consequence of aging. However, the COVID-19 pandemic has brought the issue of appetite to the frontline of the clinical care scenario, because the loss of appetite is a relevant symptom of COVID-19 (14–18), particularly in older people (16, 18).

On September 27th 2021, the International Conference on Frailty and Sarcopenia Research (ICFSR) Task Force met virtually to discuss issues related to appetite loss and aging. In particular, the implementation and management of appetite loss in at-risk older adult populations, energy balance during aging, and the design of future clinical trials on the anorexia of aging were discussed.

## Poor appetite and frailty

As a core element of nutritional status in older adulthood, loss of appetite plays a significant role in the frailty syndrome. Frailty is considered as a medical syndrome of multidimensional etiology and characterized by decreased physiological reserves (eg, reduced strength, endurance) to combat internal and external stressors. Consequently, frailty increases the individual’s vulnerability to adverse health events (19, 20), including functional loss, dependency and death. Although frailty has been historically operationalized using different models (eg, syndromic or deficits accumulation), the nutritional status has constantly been included in most operational definitions and assessment tools (such as the frailty phenotype (21) and the FRAIL scale (19)).

As it is the case for frailty, appetite control is also influenced by several determinants at different levels (1), encompassing physiological factors, pathological determinants (eg, depression, dementia, medications, poor dentition and swallowing, and other chronic diseases), and even social determinants (eg, loneliness, poverty, food insecurity). Indeed, physiological changes in appetite appear to start relatively early in old age and may act synergistically with social or health-related issues to precipitate weight loss in later years (22). Such a complex network may be illustrated using the example of the biological mechanisms involved in appetite control, with both stimulatory and inhibitory pathways from several organs/systems, such as the brain, oro-sensory signaling, muscle, gut, adipose tissues and pancreas (23). An imbalance in the causes/determinants of appetite may lead to the overexpression of anorexic pathways, potentially reducing both the quantity of caloric intake and the quality of specific nutrients (eg, proteins). Therefore, a vicious cycle can occur, with loss of appetite contributing to undernutrition of the older person, further increasing the severity of frailty.

Raising awareness around the multidimensional causes of loss of appetite and its interaction with frailty is crucial for the care of older adults in the current context of social restrictions and containments imposed by the COVID-19 pandemic. Indeed, these social isolation measures can have deleterious effects on both anorexia of aging and frailty, alongside other conditions such as sarcopenia (24). They can accentuate the above-mentioned vicious cycle through different mechanisms, including (but not limited to) increased loneliness and anxiety, reduced physical activity, changes in dietary patterns (eg, meal frequency, increased intake of processed food), and body composition changes (eg, muscle loss and increase in adipose tissue).

## Poor appetite in the ICOPE program

The World Health Organization (WHO) has recently recognized the importance of nutrition, and appetite loss as a central element of nutritional health, for the care of older individuals by introducing nutritional status as a core measure in the Integrated Care for Older People (ICOPE) program (25, 26). The ICOPE program represents a change in the care paradigm of older adults, replacing the traditional single disease-centered approach by a function- and person-centered healthcare pathway. The conceptual framework that constitutes the foundations of the ICOPE program has been presented in the World Report on Ageing and Health, published in 2015 (27), further emphasized in a subsequent publication (28). It involves the concepts of healthy aging, functional ability and intrinsic capacity. In short, healthy aging is not defined by the absence of diseases, but as the maintenance of optimal levels of functional ability that enable well-being. Functional ability is made of intrinsic capacity, the environment and their interactions; it represents the individual’s ability to do and to be what they have reason to value (ie, cope in daily life). Differently, intrinsic capacity is the composite of all the physical and mental capacities of an individual and is linked to individual’s body functions. In this context, five main body functions/domains of intrinsic capacity have been proposed, resulting in six operational domains: cognitive function, locomotion, psychological, hearing and vision (which composes the sensory domain), and vitality. It is the vitality domain that is currently operationalized through the assessment of nutritional status, with both appetite and weight loss as the conditions used to screen people at risk for malnutrition.

The ICOPE program has started to be implemented in different clinical settings and countries around the world. In the Toulouse area (Région Occitanie, France), primary care providers (29), in particular community nurses, are implementing the ICOPE program with more than 11,000 people already screened and being currently followed-up over time for detecting potential deficits in intrinsic capacity, what will inform the development of timely interventions before the onset of dependence. Initial data of the Toulouse implementation experience, obtained by March 2021, for the first 4,546 individuals, aged 60 years-old or over (mean age 78.3 years ±8.6; 61.7% women), evaluated by ICOPE are presented in Table 1.

**Table 1:** Deficit in the domains of intrinsic capacity according to the ICOPE screening tool† in 4,546 older adults in the Toulouse area (Région Occitanie, France) — data obtained by up to March 2021

**Deficit in intrinsic capacity**	Total (n=4546)*	**Loss of appetite‡**	
		**Yes (n=704)**	**No (n=3842)**
Cognition^a^	2765 (60.8%)	562 (79.8%)	2203 (57.3%)
Locomotion^b^	1781 (39.2%)	426 (60.5%)	1355 (35.3%)
Psychological^c^	1767 (38.9%)	504 (71.6%)	1263 (32.9%)
Vision^d^	3310 (72.8%)	530 (75.3%)	2780 (72.3%)
Hearing^e^	2462 (54.1%)	439 (62.3%)	2023 (52.6%)
Vitality^f^ (n=4,529)	988 (21.8%)	699 (100%)	289 (7%)

*Population may vary due to missing data; ‡Percentages reflect the prevalence of deficits in intrinsic capacity within the groups with and without loss of appetite; †Operational definitions of deficits in intrinsic capacity domains according to the ICOPE screening tool are thoroughly described elsewhere (25) and summarized below (letters a to f); a. Cognition deficit: any wrong response to the 3-word recall or time and space orientation; b. Locomotion deficit: Inability to rise from a chair five times without using arms in ≤ 14 seconds; c. Psychological deficit: Responding “Yes” to any of the following two questions “Over the past two weeks, have you been bothered by: 1. Feeling down, depressed or hopeless? 2. Little interest or pleasure in doing things?”; d. Vision deficit: Responding “Yes” to the following question “Do you have any problems with your eyes: difficulties in seeing far, reading, eye diseases or currently under medical treatment (e.g. diabetes, high blood pressure)?”; e. Hearing deficit: Deficit in any of the right or left ears measured through hears whispers (whisper test); f. Vitality deficit: Responding “Yes” to any of the following two questions “1. Weight loss: Have you unintentionally lost more than 3kg over the last three months? 2. Appetite loss: Have you experienced loss of appetite?”

Beyond the data displayed on Table 1, the Toulouse dataset showed that only 45.1% (n=315) of people with appetite loss also had unintentional weight loss; the prevalence of weight loss in this sample was 13.3% (n=604). Binary logistic regressions adjusted by age and sex showed an increased probability (all p-values < 0.05) of deficits in intrinsic capacity among individuals experiencing appetite loss, compared to those without appetite loss: Cognition deficit. OR=2.45, 95% CI 2.01 – 2.99; Locomotion deficit. OR=2.24, 95% CI 1.88 – 2.66; Psychological deficit. OR=4.82, 95% CI 4.02 – 5.77; Vision deficit. OR=1.23, 95% CI 1.02 – 1.48; Hearing deficit. OR=1.22, 95% CI 1.07 – 1.50. These data support the importance of appetite loss not only for determining nutritional status, but also in a more global perspective for maintaining optimal functional levels as people age. The management of appetite loss appears thus to be crucial in a healthy aging perspective.

The ICOPE program is designed to screen, assess and treat deficits in intrinsic capacity in order to keep functional ability for as long as possible, thus preventing or delaying the onset of disabilities and care dependence. ICOPE and its guiding line model of intrinsic capacity have been released only recently. Evolutions in founding concepts, particularly regarding the construct of vitality (for which current measure is operationalized by nutritional status), are being proposed(30), which could modify the operational measurements used in the future. In this context, it is the opinion of the members of the ICFSR Task Force that the importance of appetite loss and more globally nutritional status must be reinforced as a core evaluation to be undertaken in any care model devoted to older people.

## Declining energy requirements and implications for nutritional health in old age

Nutritional health represents a dynamic process throughout the life span. According to recent data using the gold standard assessment of energy expenditure double-labelled water (DLW), the human organism requires more energy in the development phases during childhood, followed by a decline up to around 30 years-old, when a plateau is observed until 60 years-old, and then a further progressive decline is observed in late ages (31). Although there is no consensual data from longitudinal DLW studies, it seems that energy requirements decline by 17%–25% between the ages of 20 years- and 80-years-old. Such a decline may be explained by a reduced energy expenditure for both physical activity (32) and basal energy expenditure together with an impairment in the thermic response to food during aging (33). This overall reduction in energy requirements during aging, alongside the common condition of appetite loss, probably increases the risk for older adults not meeting the recommended amounts of healthy food groups as well as recommended amounts of specific essential nutrients. This may lead to malnutrition/undernutrition and the vicious cycle with frailty. Overall, it is complicated to respect dietary recommendations in older adults consuming less than 1600kcal/day, and essentially impossible for diets at 1000kcal/day(34).

There are several gaps in the field of energy expenditure during aging: first, very few reliable data on energy expenditure through the life course is currently available; this lack of data is still more pronounced for people over 80 years-old. As a consequence, there is an absence of normative data on energy expenditure in older adulthood. Second, studies on DLW have mostly relied on cross-sectional data. Longitudinal data is therefore needed in order to better control for interindividual variability, which is much important during aging. Investigations on energy expenditure during aging are crucial to inform the development of evidence-based nutritional recommendations for older people, with potential implications for research (for example, on caloric restriction, a non-pharmacological intervention that has the potential to slow down the rates of biological aging, but data is still lacking for older people) and clinical practice.

## Anorexia of aging: Assessment and Management

Anorexia of aging (ie, loss of appetite and/or low food intake in older people), as all age-related conditions including frailty, shares biological and physiological alterations partly driven by the aging process, with particular importance to oral/sensory health (eg, dry mouth, reduced taste/smell, reduced thirst), gastrointestinal function (eg, altered motility, with reduction of stomach compliance and delayed gastric empty) and body composition changes (eg, reduction in muscle mass and bone density, increase in fat mass, with repercussions on both physiological [eg, decreased basal metabolic rate, strength] and functional [eg, swallowing problems] capacities); this latter constituting one of the core components of the aging phenotype (35, 36). As most geriatric syndromes, the determinants of anorexia go far beyond the simple succession of biological cascades and physiological processes, with major determinants related to psychological (eg, loneliness, fatigue, depression), social (eg, sex roles), cultural (eg, culture of lean body), and even economical (eg, limited income) aspects. Table 2 displays some important determinants of anorexia of aging, the potential at-risk populations that should be closely assessed and monitored for anorexia, as well as the nutritional strategies that can make part of clinical care.

**Table 2:** Anorexia of aging: determinants, at-risk populations and nutritional strategies against malnutrition

**Determinants**	**At-risk populations**	**Nutritional strategies**
*Physiological/Functional* Swallowing/chewing problems Poor dentition Dry mouth Reduced thirst/dehydration Reduced taste/smell Altered gastrointestinal motility Decreased stomach compliance Delayed gastric emptying Body composition changes Reduction in muscle mass & basal metabolic rate *Comorbidities/conditions* Neurological diseases (eg, dementia) Gastrointestinal diseases Depression Fatigue Sarcopenia Inflammatory conditions Disability (eg, chair/bed bound) Drugs *Psycho-social* Loneliness/social isolation Socio-economic deprivation/Poverty *Others* Lack of cooking skills Low food variety Cultural (eg, lean body culture, especially for women)	Hospitalized Institutionalized (eg, nursing homes) People living alone Multimorbid and disabled	Modify diet prescription Food fortification Vitamin/mineral supplements Oral nutritional supplements (monitor the gap between prescription and intake) Exercise — Progressive Resistance Training

Anorexia can be seen as an early sign of an evolving malnutrition condition, being a symptom that, in most cases, would precede weight loss(37). However, even though it is possible that anorexia and weight loss make part of the same condition, ie, malnutrition, with different time-arousal phenotypic expressions, the deleterious consequences of anorexia are independent of, although potentiated by weight loss. Indeed, anorexia was associated with an increased 83% risk of 10-month mortality among community-dwelling older adults receiving home care (38); although mortality risk was higher in people with both anorexia and weight loss, individuals with anorexia and no weight loss had an increased mortality risk of 45%, compared to subjects without anorexia. Moreover, another study suggested that individuals with anorexia alone or anorexia combined with weight loss had similar results in performance-based tests of mobility (eg, walking speed), but these subjects with anorexia (independently of their weight loss status) had worse performance in mobility tests compared to people without anorexia (39).

Therefore, the lack of energy driven by anorexia results in poor physical performance and adverse health events in the elderly; these associations are in part independent of other clinical conditions. The assessment and management of anorexia of aging, and more globally of malnutrition, must be taken into account in routine clinical practice among geriatric patients. There are simple scales assessing anorexia that can be appropriate for clinical use, such as the Functional Assessment of Anorexia/Cachexia Therapy (FAACT) and the Simplified Nutritional Assessment Questionnaire (SNAQ). To date, drugs to combat anorexia of aging (eg, corticosteroids, growth hormone, anabolic steroids, metoclopramide, and appetite-stimulating drugs [eg, megesterol, meclobemide…]) are still of limited utility in clinical practice mostly due to an unfavorable risk/benefit balance. Therefore, other nutritional interventions (as exemplified in Table 2) should be privileged.

## Anorexia drug trials design

As indicated in the previous section of this manuscript, strategies to treat anorexia of aging in clinical practice are currently very limited, targeting mostly malnutrition (not specifically targeting anorexia), without useful drugs that clearly present a favorable risk/benefit ratio. Given the prevalence and burden of anorexia of aging (including an increased risk of weight loss and malnutrition), efforts to develop effective pharmacological interventions to combat this condition, which may ultimately prevent and decrease the severity of malnutrition, should be stimulated and pursued. In this context, it is of utmost importance that trialists define the essential methodological aspects for a study aiming at testing the effects of a molecular agent on appetite loss/anorexia. This represents a major challenge given the multifactorial etiology of anorexia and the multidimensional nature of its contributors. Indeed, due to this multitude of etiological factors and determinants, several types of appetite loss may exist under the umbrella term of anorexia of aging. For instance, providing an appetite-stimulating medication to a person with depression-dependent anorexia is arguable since the origin of the syndrome is not addressed. On the other hand, subdividing anorexia into various subtypes will complexify the study design and may render the development of future investigations unreasonable and almost impossible to be achieved due to issues such as statistical study power (sample size will be larger if the study must account for the effects of a drug on each anorexia subtype) and consequent economic burden. One potential intermediate solution is to have two main anorexia subtypes: 1) anorexia with associated cachexia and 2) anorexia without cachexia. Future trials might target one of these two subtypes or ideally be powered to address both. Besides the difficulty of defining the best target population (inclusion/exclusion criteria), other challenges that must be solved involve defining: the most appropriate assessment tool for appetite, the pre-planned primary/secondary outcome measures, and study length. Although there are short and simple questionnaires to measure appetite in older adults with good sensitivity and specificity to detect weight loss, such as the 8-item Council on Nutrition appetite questionnaire (CNAQ) or its 4-item derivative SNAQ(40), no gold standard measurement exists. Regarding outcome measures, although some endpoints must indisputably make part of the pre-specified outcomes, such as appetite and weight, other outcomes (eg, mobility, muscle strength/sarcopenia) may increase the reach of the trial and be necessary for future approval by regulatory agencies. Furthermore, it is likely that trials should consider a 3 to 12 months of follow-up, according to the trial phase. This timeframe may be required for providing the intervention with enough time for producing effects on the primary, but also on key secondary outcomes. Table 3 summarizes the essential elements to be considered when designing future drug trials targeting appetite loss/anorexia of aging.

**Table 3:** Designing future drug trials targeting appetite loss/anorexia of aging

**Target population**	**Assessment tool of appetite/anorexia**	**Outcome measures**	**Study length**
*Inclusion criteria* ≥ 65 years-old Chronic appetite loss (avoid people with acute loss due to infections or others) Reduced food intake *Other conditional inclusion criteria* Chronic on-going weight loss *Exclusion criteria* Depression Severe cognitive impairment Dysphagia, dental or oral problems	SNAQ FAACT CNAC Other	Primary Appetite Weight Secondary Physical performance/mobility Muscle strength Quality of life Survival Body composition (eg, lean/fat mass)	Between 3 months (for phase 2 trials) and 12 months (for phase 3 trials, with more difficult-to-change clinical outcomes, such as mobility)

## Conclusions

Loss of appetite/anorexia of aging is a prevalent and burdensome geriatric syndrome. Yet, it is under-evaluated and, as a consequence, under-diagnosed and under-treated in routine clinical practice. Future actions in this field should focus on the systematic assessment of appetite loss in the care pathway of older people, as it is the case for the ICOPE program launched by WHO. Moreover, clinical care should move from assessment to the treatment of this syndrome, whereas researchers pursue their efforts to find effective pharmacologic and non-pharmacologic interventions with a favorable risk/benefit ratio.
